# Isoniazid MIC and *Kat*G Gene Mutations among *Mycobacterium tuberculosis* Isolates in Northwest of Iran

**Published:** 2011

**Authors:** Seed Reza Moaddab, Safar Farajnia, Davood Kardan, Sajad Zamanlou, Mohammad Yousef Alikhani

**Affiliations:** 1 *Tuberculosis and Lung Research Centre and Paramedical Faculty, Tabriz University of Medical Sciences, Tabriz, Iran*; 2 *Biotechnology Research Centre, Tabriz University of Medical Sciences, Tabriz, Iran*; 3 *Infectious and Tropical Disease Research Centre, Tabriz University of Medical Sciences, Tabriz, Iran*; 4 *Immunology Research Center, Tabriz University of Medical Sciences, Tabriz, Iran*; 5 *Microbiology department, Faculty of Medicine, Hamadan University of Medical Sciences, Hamadan, Iran*

**Keywords:** Isoniazid Resistance, Kat G, Mycobacterium tuberculosis, PCR, RFLP

## Abstract

**Objective(s):**

Isoniazid (INH) is one of the main first line drugs used in treatment of tuberculosis and development of resistance against this compound can result in serious problems in treatment procedures. Resistance to INH is mediated mainly by mutation in *Kat*G gene that is coded for the catalase enzyme. The proportional method for detection of INH-resistance is time consuming due to the slow growth rate of *Mycobacterium tuberculosis*. In this study, we used PCR-RFLP approach for screening of common mutations in *Kat*G gene for detection of INH resistance, and compared the results to minimal inhibitory concentration (MIC) in *M. tuberculosis* isolates.

**Materials and Methods:**

Fifty *M. tuberculosis* isolates were subjected to study of which, 25 strains were INH-resistant and 25 strains were INH-sensitive.

**Results:**

Of 25 INH-resistant strains, the mutation was identified in 56% and 20% in the *Kat*G315 and *Kat*G463 loci, respectively. In 24% of INH-resistant strains, no mutation was observed in the studied loci. INH MIC was <0.2 μg/ml in all sensitive strains whereas among 25 INH -resistant isolates, INH MIC was higer than 0.2 μg/ml ranged from 0.2 to 3.2 μg/ml.

**Conclusion:**

Our findings revealed that PCR-RFLP is capable to identify INH-resistance in more than 76% of INH- resistant* M. tuberculosis* strains, and could be used for rapid identification of INH resistance. High levels of INH MIC were observed in the strains which had mutation in the *Kat*G gene in position 315.

## Introduction

Although tuberculosis is regarded as a historical disease, in the recent years, the medical community has increasingly worried about the spread of drug resistant *Mycobacterium tuberculosis *strains. Isoniazid (INH) and rifampin are two first line drugs against tuberculosis and strains display resistance against these drugs are considered as multi drug Resistant (MDR) *M. tuberculosis* (1-3). The control program of tuberculosis has been affected seriously by the spread of MDR strains and so, rapid identification of resistant strains has a special impact on the treatment and control of dissemination of MDR strains in the communities (4,5). INH is a pro-drug that enters into the bacillus via passive diffusion, changes into an active form by enzyme catalase-peroxidase, and then the free radicals attack different targets in the microorganism. The previous observations have showed that tuberculosis bacillus lose their acid fastness property after exposure to INH (6-9).

Drug resistance in bacterial population takes place randomly by mutations. Resistance to INH is associated with a variety of mutations in several genes like *Kat*G, *inh*A, *oxy*R-*aph*C, *kas*A and *ndh *(4, 8,10). The *Kat*G gene encodes enzyme catalase-peroxidase and mutation in this gene decreases or blocks the enzyme activity. Mutation in the *kat*G gene is the main mechanism of INH-resistance in most strains (11).

There are various methods for detection of INH-resistant* M. tuberculosis *strains including conventional and molecular methods. The conventional methods based on culture and proportional assay of the bacterial sensitivity are time consuming and need several weeks for bacterial growth. In the recent years, rapid molecular techniques such as PCR-RFLP (polymerase chain reaction-restriction fragment length polymorphism) have been proposed for detection of resistance to INH. This method reduces the time of diagnosis to few hours (1, 3, 4,11). Since the frequency and type of mutations in the *Kat*G gene are varied in different geographic regions, this study aimed to determine the prevalence of mutations in the *Kat*G gene and INH minimal inhibition concentration (MIC) in* M. tuberculosis *strains in Northwest of Iran.

## Materials and Methods

The bacteria included in this study were 50 isolates of *M. tuberculosis *which were recovered from pulmonary tuberculosis patients. Out of 50 strains, 25 were susceptible and 25 were resistant to INH. Bacterial culture was accomplished in Lowenstein-Jensen (LJ) medium and the isolates were identified by standard microbiological methods such as Ziehl-Neelson staining, morphology of colony, pigment and niacin production, nitrate and catalase tests. For susceptibility testing the proportional method was employed (12-14). The MICs of INH (0.2, 0.4, 0.8, 1.6 and 3.2 μg/ml) was determined on LJ medium for INH- resistant strains. For all experiments, *M. tuberculosis* HRV37 was used as standard.

***DNA Extraction***

For bacterial DNA extraction, the grown colonies of *M. tuberculosis* on LJ medium was resuspended in 300 µl TE buffer (Tris 10 mM, pH 8; EDTA 1 mM) and inactivated for 40 minutes at 80 °C. Then the DNA of isolates was extracted by SDS and proteinase-K method (4,15). 

***PCR-RFLP***

The coding region of *Kat*G gene was amplified by primers *Kat*G 904: 5′-AGCTCGTATGGC ACCGGAAC3′ and *Kat*G 1523: 5′-TTGA CCTCCCACCCGACTTG3′ as described previously (16). The PCR reactions was prepared in a 25 µl volume included 10 pM of primers (1 µl of each forward and reverse primers), 2 mM MgCl2 (1.5 µl), 0.2 mM dNTPs (0.5 µl) and 2 unit (0.25 µl) of Taq DNA polymerase (Fermentas). The PCR program was initial denaturation at 94 °C for 4 minutes followed by 35 cycles of denaturation at 94 °C for 1 min, annealing at 63 °C for 1min and extension at 72 °C for 1 min with a final extension at 72 °C for 5 min. In order to characterize the mutations in the *Kat*G gene, the PCR products were purified with PCR product purification columns () and digested by Mspl restriction enzyme (Fermentas). Finally, the reaction mixture was electrophoresed on 1% agarose gel and visualized on UV light after staining with ethidium bromide (16,17).

## Results

Totally, 50* M. tuberculosis *strains were isolated from pulmonary tuberculosis patients among which 28 (56%) were males and 22 (44%) were females. The age range of patients was 23 to 63 years and the median age was 42.92 years. Out of 50 *M. tuberculosis *strains, 25 strains were resistant and 25 strains were found to be sensitive to INH by proportional method. We studied the INH MIC among 25 resistant isolates and we also aimed to determine the probability of relationship between MIC ranges and type of mutations in the *Kat*G gene. According to the protocol of proportional method, strains with MIC 0.2 μg/ml and higher was considered as INH resistant. The results indicated that among 25 INH-resistant strains, the MICs were 0.4 μg/ml in one (4%) isolate, 0.8 μg/ml in one (4%) isolate, 1.6 μg/ml in 3 (12%) isolates and 3.2 μg/ml in 4 (16 %) isolates. In the remaining 16 (64%) resistant isolates, the MICs were 0.2 μg/ml. The MICs of *M. tuberculosis* strain HRV37 (standard strain) and all INH-sensitive isolates, were less than 0.2 μg/ml. 

**Figure 1.**
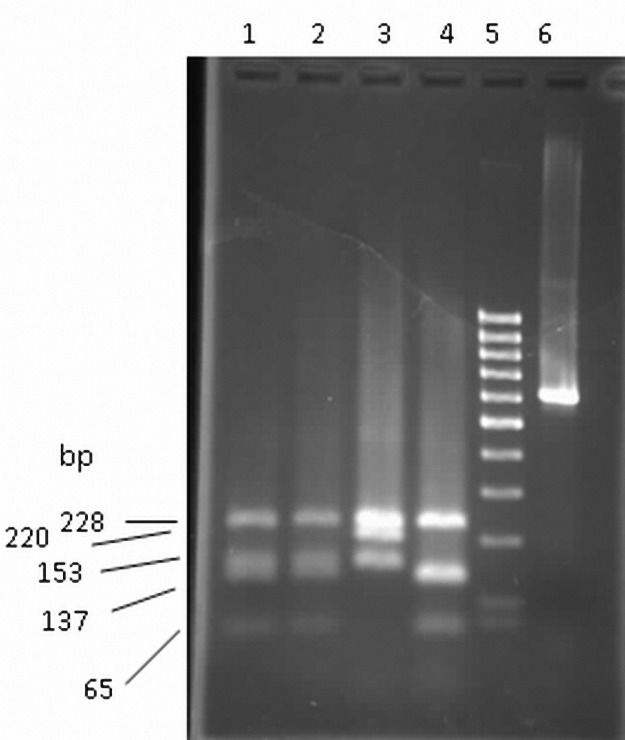
PCR RFLP pattern of INH-sensitive and INH-resistant *M.tuberclosis* isolates. Lanes 1 and 2, INH sensitive wild type; lane 3, R-463-L mutant; lane 4, S-315-T mutant isolates and lane 6, PCR amplified *Kat*G gene before restriction digestion. Lane 5 is related a 100 bp DNA ladder.

PCR amplification of *Kat*G gene was positive in all isolates and resulted in a 620 bp product in agarose gel electrophoresis. After Msp1 digestion of *Kat*G amplicon, four distinct RFLP patterns were observed (16) (Table  and  Figure 1).

Our findings showed that 25 INH-sensitive *M. tuberculosis *strains had no mutation in the *Kat*G gene. Among 25 INH-resistant isolates, 14 strains (56%) showed mutation in the *Kat*G315 and 5 strains (20%) showed mutation in the *Kat*G 463 loci. Six resistant isolates (24%) had no mutation in the studied loci. Mutation in both 315 and 463 codons were not found in any isolates. *M. tuberculosis* strain HRV37 susceptible to all anti-tuberculosis drugs was used for quality control. This strain was negative for mutation in the* Kat*G gene in PCR-RFLP. For confirmation of RFLP results, the PCR products from 10 stains were submitted for sequencing (MWG, ). The sequencing results were in agreement with PCR-RFLP results for both INH-resistant and INH-sensitive strains (Table 2). 

**Table1.** Predicted fragment sizes of Msp1 digest of *Kat*G amplicone.

KatG mutation	DNA fragments (bp)					
Wild type	65		137	153		228
at codon 315 (S→T)	65	132	137			228
at codon 463 (R→L)				153	220	228
at codons 315 and 463		132			220	228

**Table 2.** DNA fragments sizes of PCR products after digestion with Mspl.

Mutation	No. of strains	Fragments (bp)						Observed phenotype
		65	132	137	153	220	228	
No mutation	31 (6 INH resistant, and 25 INH sensitive isolates)	+		+	+		+	Wild type
At codon 315	14	+	+	+			+	Type 1
At codon 463	5				+	+	+	Type 2

## Discussion

The spread of INH and rifampin resistant *M. tuberculosis* strains, which are known as MDR strains, have caused a major health problem in global community (18). Rapid detection of MDR strains would help efficient treatment of patients and prevents widespread distribution of these isolates (1-5). This study aimed to survey the use of PCR-RFLP in comparison with proportional method for rapid detection of INH resistant* M. tuberculosis* strains. 

The resistance to INH was screened by proportional method in LJ medium and then INH MIC was determined in INH-resistant isolates. Analysis of mutation in two *Kat*G gene loci was carried out by PCR- RFLP. The results indicated that PCR-RFLP was able to detect INH- resistance in 76% of 25 resistant strains. Mutation was identified at codon 315 in 56% of strains whereas codon 463 contained mutation in 20% of resistant strains. 

Twenty five INH- sensitive *M. tuberculosis* strains as well as standard strain (*M. tuberculosis* H37RV) showed no mutation in the studied codons. These findings are consistent with some reports about the rate of mutation in the *Kat*G gene in other parts of . Zaker Bostanabad *et al* (19) reported that 61% of INH-resistant *M. tuberculosis* isolates in the east region of contained mutation in the *Kat*G gene. In another study carried out on the strains of Isfahan and Tehran, the mutation rate was 78% for Isfahan and 61% for Tehran (20). In other countries, the results varied in different part of the world. For instance, in Turkey, Cavusoglu *et al* (21) reported the mutation rate of 73% and in another study taken place in east of Turkey; the rate of mutation in the *Kat*G was reported as 63% (22). Among countries in the southern boundary of Iran, the percentage of mutation in the *Kat*G gene was reported 65% in Kuwait, 64% in Dubai, 35% in Beirut and 47% in Egypt (23-26). In the other countries, the mutations in the *Kat*G have been reported as 98% in Russia, 84% in Lithonia, 71% in Vietnam, 65% in Australia, 60% in South Africa, 60% in China, 60% in Philippines, 59% in Spain, 46% in Switzerland, and 55% in India (1, 3, 21, 25,26).

In the present study, 20% of INH- resistant strains had mutation in the *Kat*G codon 463 and no simultaneous mutation was observed in either codons 315 and 463. Zhang *et al* (27) reported mutation at codon 463 in 40% of INH-resistant *M. tuberculosis* strains. In 24% of our INH-resistant strains (6 strains) no mutation was identified in the *Kat*G gene in the study loci. In a study in India, in 27% of 120 INH-resistant strains, no mutation was detected in the *Kat*G gene (4). These findings show that mutations in other codons of *Kat*G gene or mutations in other genes such as *inh*A, *oxy*R-*aph*C, *kas*A and *ndh *might be involved in INH-resistance in these isolates (3, 4, 8, 19, 23,25).

Analysis of INH MIC among 25 INH-resistant strains demonstrated that the MIC was 0.2 μg/ml in 16 strains, 0.4 μg/ml in one strain, 1.6 μg/ml in 3 strains, and 3.2 μg/ml in 4 strains. The results also indicated that the MIC of most strains with mutation at codon 315 was >2 μg/ml. These findings are consistent with reports on correlation of INH MIC and type of mutation in *Kat*G gene (28-31). It has been reported that the level of INH MIC is correlated to the type and extent of changes in the *Kat*G gene. Hass *et al*, (28) reported INH MIC>50 μg/ml in the strains which have lost their *Kat*G totally on their chromosome whereas, in 83% of INH- resistant strains with mutation at codon 315, the INH MIC have been >2 μg/ml. Our findings in accordance with previous results showed that minor changes in the *Kat*G gene were associated with decreased catalase activity and low INH MIC (28). 

## Conclusion

The result of this study revealed that mutation at codons 315 and 463 of *Kat*G gene is responsible for INH resistance in 76% of *M. tuberculosis* strains in northwestern of . The result also indicated that PCR-RFLP method can be used for rapid diagnosis of NIH-resistant tuberculosis in most cases. 
